# Supplementary material for: Analysis of DNA methylation associates the cystine–glutamate antiporter SLC7A11 with risk of Parkinson’s disease
Source: Nat Commun. 2020 Mar 6;11:1238. doi: 10.1038/s41467-020-15065-7 (PMC7060318; doi:10.1038/s41467-020-15065-7)
Supplement: Supplementary file 1 — Supplementary Information [file 41467_2020_15065_MOESM1_ESM.pdf]

Supplementary Information

Analysis of DNA methylation associates the cystine-glutamate antiporter *SLC7A11* with risk of Parkinson's disease

Costanza L. Vallerga[1], Futao Zhang[1], Javed Fowdar[2], Allan F. McRae[1], Ting Qi[1], Marta F. Nabais[1], Qian Zhang[1], Irfahan Kassam[1], Anjali K. Henders[1], Leanne Wallace[1], Grant Montgomery[1], Yu-Hsuan Chuang[3], Steve Horvath[4,5], Beate Ritz[3,6,7], Glenda Halliday[8], Ian Hickie[8], John B. Kwok[8], John Pearson[9], Toni Pitcher[10,11], Martin Kennedy[9], Steven Bentley[2], Peter A. Silburn[12], Jian Yang[1,12], Naomi R. Wray[1,12], Simon Lewis[8], Tim Anderson[10,11], John Dairymple-Alford[10,13], George D. Mellick[2,*], Peter M. Visscher[1,12,*,#], Jacob Gratten[14,1,*,#]

[1] *Institute for Molecular Bioscience, The University of Queensland, Brisbane, Australia*

[2] *Griffith Institute for Drug Discovery (GRIDD), Griffith University, Brisbane, Australia*

[3] *Department of Epidemiology, Fielding School of Public Health, UCLA, Los Angeles, CA, USA.*

[4] *Department of Human Genetics, David Geffen School of Medicine, University of California Los Angeles (UCLA), Los Angeles, CA, USA*

[5] *Department of Biostatistics, Fielding School of Public Health, UCLA, Los Angeles, CA, USA*

[6] *Department of Neurology, David Geffen School of Medicine, UCLA, Los Angeles, CA, USA.*

[7] *Department of Environmental Health, Fielding School of Public Health, UCLA, Los Angeles, CA, USA.*

[8] *Brain and Mind Centre, Sydney Medical School, The University of Sydney, Sydney, Australia*

[9] *Department of Pathology, University of Otago, Christchurch, New Zealand*

[10] *New Zealand Brain Research Institute, Christchurch, New Zealand*

[11] *Department of Medicine, University of Otago, Christchurch, New Zealand*

[12] *Queensland Brain Institute, The University of Queensland, Brisbane, Australia*

[13] *Department of Psychology, University of Canterbury, Christchurch, New Zealand*

[14] *Mater Research Institute, The University of Queensland, Brisbane, Australia*

[*] *These authors contributed equally to this work*

[#] *Corresponding authors:* jacob.gratten@mater.uq.edu.au; peter.visscher@uq.edu.au

Supplementary note

List of abbreviations in the main text.

ALS/PDC:	Amyotrophic lateral sclerosis/parkinsonism-dementia complex
AUC:	Area under the receiver operator characteristic curve
BMAA:	β-methylamino-L-alanine
CTPs:	Cell type proportions
PEG:	Parkinson’s disease, Environment and Genes case-control cohort
MOA:	Mixed linear model-based omic association
MOMENT:	Multi component mixed linear model-based omic association excluding the target
MWAS:	Methylome-wide association study
OREML:	Omics restricted maximum likelihood
OSCA:	OmicsS-data-based Complex trait Analysis
PD:	Parkinson’s disease
SGPD:	Systems Genomics of Parkinson’s Disease consortium

Supplementary Table 1: Characteristics of the SGPD case-control cohort.

**Characteristics**	**SGPD (N_{Tot} = 1638)**	**Effective N**
PD diagnostic status, N (%)	851 (52%)	1638
Predicted age, Mean (range)	70.05 (26-93)	1638
Sex, Male (%)	925 (56%)	1638
Sex controls, Male (%)	386 (49%)	787
Sex PD diagnostic status, Male (%)	539 (63%)	851
Ever cigarette smoking, N (%)	784 (53%)	1487
Ever coffee consumption, N (%)	949 (77%)	1239
Ever exposure to pesticides, N (%)	262 (18%)	1475
Ever farm work, N (%)	425 (34%)	1251
Ever welding, N (%)	91 (7%)	1251
Levodopa daily dose (mg/day) in PD cases, Mean (SD)	866.63 (815.41)	494

Supplementary Table 2: Summary of the case-control differences in cell type proportions for cases versus controls, the most highly medication-exposed cases (top 10% of LEDD, N = 50) versus controls, and the least medication-exposed cases (bottom 10% of LEDD, N = 50) versus controls. Granulocytes are calculated as the sum of eosinophil and neutrophil proportions. NK, natural killer cells; SD, standard deviation; Mean diff*, mean different between case group and controls.

**Cell type**	**Controls**		**PD cases (N = 494)**			**Cases: bottom 10% LEDD**			**Cases: top 10% LEDD**		
	**Mean**	**SD**	**Mean**	**SD**	**Mean diff***	**Mean**	**SD**	**Mean diff***	**Mean**	**SD**	**Mean diff***
Bcell	0.051	0.043	0.039	0.055	0.012	0.036	0.016	0.015	0.040	0.028	0.011
CD4T	0.159	0.071	0.135	0.068	0.024	0.155	0.061	0.004	0.132	0.065	0.027
CD8T	0.049	0.050	0.045	0.047	0.004	0.063	0.055	-0.014	0.040	0.039	0.009
Eosinophils	$1.5x10^{-03}$	0.009	$1.1x10^{-03}$	0.008	$4x10^{-04}$	$-3.3x10^{-19}$	$4.4x10^{-18}$	0.002	0.001	0.005	0.001
Monocytes	0.091	0.024	0.091	0.025	0.000	0.088	0.022	0.003	0.093	0.026	-0.002
Neutrophils	0.561	0.123	0.604	0.129	-0.043	0.563	0.100	-0.002	0.599	0.128	-0.038
NK	0.117	0.055	0.112	0.053	0.005	0.121	0.042	-0.004	0.123	0.056	-0.006
Granulocytes	0.562	0.121	0.605	0.127	-0.043	0.563	0.100	-0.001	0.599	0.128	-0.037

Supplementary Table 3: Results of Summary data-based Mendelian Randomization (SMR) and HEIDI tests (b, beta; se, standard error; p, p-value) for association of cg06690548 methylation (exposure variable; "Exp") with expression at neighboring genes (outcome variable; "Out"). The analysis showed that hypermethylation of cg06690548 is associated with down-regulation of *SLC7A11*. Chr, chromosome; bp, base position; Freq, frequency; A1, allele 1; A2, allele 2; Nsnp HEIDI, number of SNPs in the HEIDI test.

ID_Out	Chr_Out	Gene_Out	bp_Out	topSNP	topSNP chr	topSNP bp	A1	A2	Freq	b_Out	se_Out	p_Out	b_Exp	se_Exp	p_Exp	b_SMR	se_SMR	p_SMR	p_HEIDI	Nsnp HEIDI
ENSG00000189184	4	PCDH18	138446860	rs6811062	4	139143747	G	A	0.920	0.069	0.122	5.74E-01	0.384	0.063	9.68E-10	0.178	0.319	5.76E-01	4.31E-01	12
ENSG00000251632	4	RP11-714L20.1	138495734	rs6811062	4	139143747	G	A	0.920	-0.036	0.142	7.99E-01	0.384	0.063	9.68E-10	-0.094	0.368	7.99E-01	3.76E-01	12
ENSG00000250033	4	SLC7A11-AS1	139038974	rs6811062	4	139143747	G	A	0.920	-0.046	0.112	6.77E-01	0.384	0.063	9.68E-10	-0.121	0.291	6.78E-01	2.72E-01	12
ENSG00000151012	4	SLC7A11	139124377	rs6811062	4	139143747	G	A	0.920	-0.306	0.092	9.27E-04	0.384	0.063	9.68E-10	-0.796	0.273	3.59E-03	1.62E-01	12
ENSG00000251372	4	LINC00499	139288181	rs6811062	4	139143747	G	A	0.920	0.156	0.100	1.19E-01	0.384	0.063	9.68E-10	0.405	0.268	1.31E-01	5.25E-01	12
ENSG00000249381	4	LINC00500	139352350	rs6811062	4	139143747	G	A	0.920	0.004	0.187	9.83E-01	0.384	0.063	9.68E-10	0.010	0.488	9.83E-01	5.90E-01	11
ENSG00000248795	4	RP11-173E2.1	139482476	rs6811062	4	139143747	G	A	0.920	-0.120	0.248	6.29E-01	0.384	0.063	9.68E-10	-0.312	0.647	6.30E-01	3.02E-01	10
ENSG00000250977	4	RP11-173E2.2	139586301	rs6849136	4	139152030	A	C	0.889	0.028	0.226	9.02E-01	0.330	0.056	3.94E-09	0.085	0.686	9.02E-01	6.85E-01	10
ENSG00000250501	4	RP11-98O2.1	139708748	rs6811062	4	139143747	G	A	0.920	0.114	0.184	5.35E-01	0.384	0.063	9.68E-10	0.296	0.481	5.37E-01	8.67E-01	10
ENSG00000151014	4	CCRN4L	139951924	rs6811062	4	139143747	G	A	0.920	-0.179	0.111	1.07E-01	0.384	0.063	9.68E-10	-0.465	0.299	1.19E-01	1.50E-01	12
ENSG00000109381	4	ELF2	140023819	rs6811062	4	139143747	G	A	0.920	0.034	0.088	6.95E-01	0.384	0.063	9.68E-10	0.089	0.229	6.96E-01	3.64E-02	12
ENSG00000179967	4	PPP1R14BP3	140036306	rs6811062	4	139143747	G	A	0.920	-0.082	0.083	3.21E-01	0.384	0.063	9.68E-10	-0.214	0.218	3.28E-01	4.19E-02	12

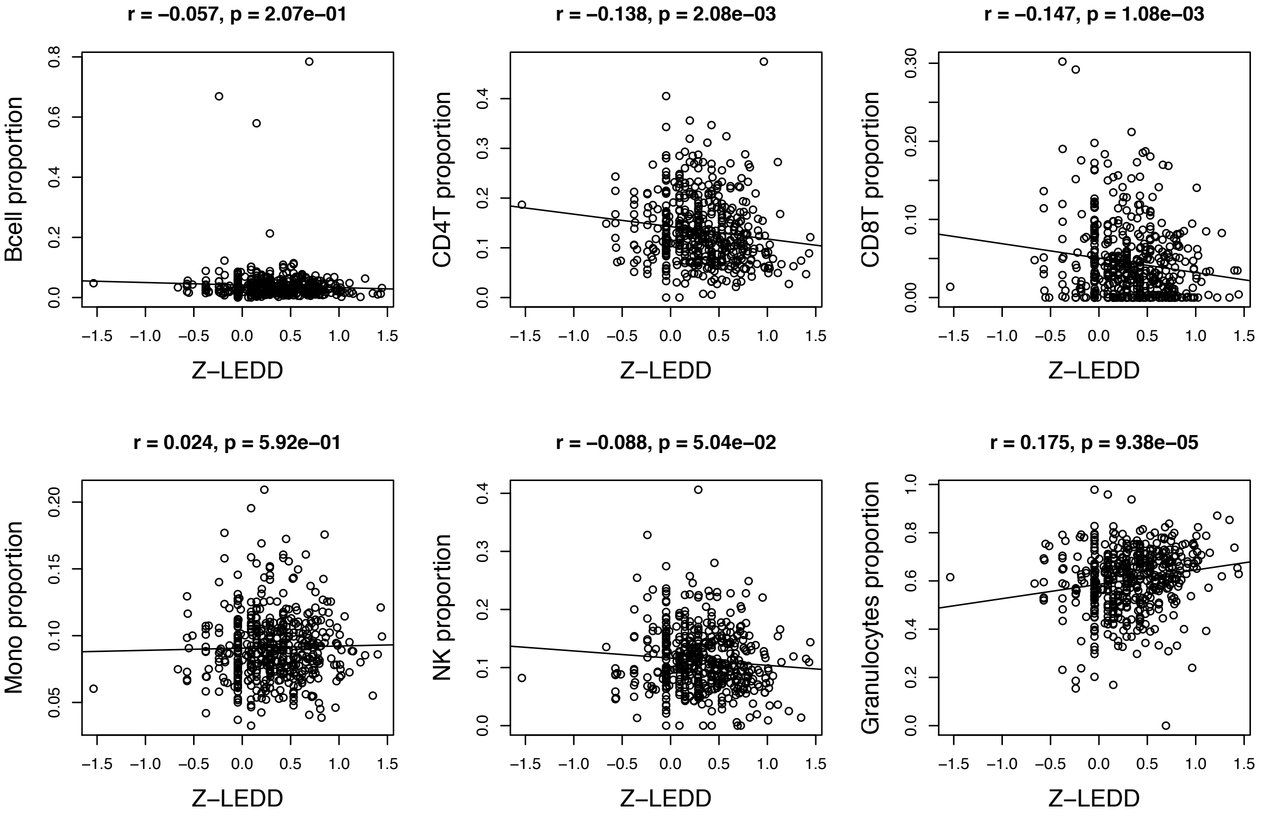

Supplementary Figure 1: Relationship (Pearson's correlation) between blood cell type proportions and Z-scaled levodopa equivalent daily dosage (LEDD) in 494 PD cases from the SGPD dataset. Granulocytes are calculated as the sum of eosinophil and neutrophil proportions. NK, natural killer cells; Mono, monocytes.

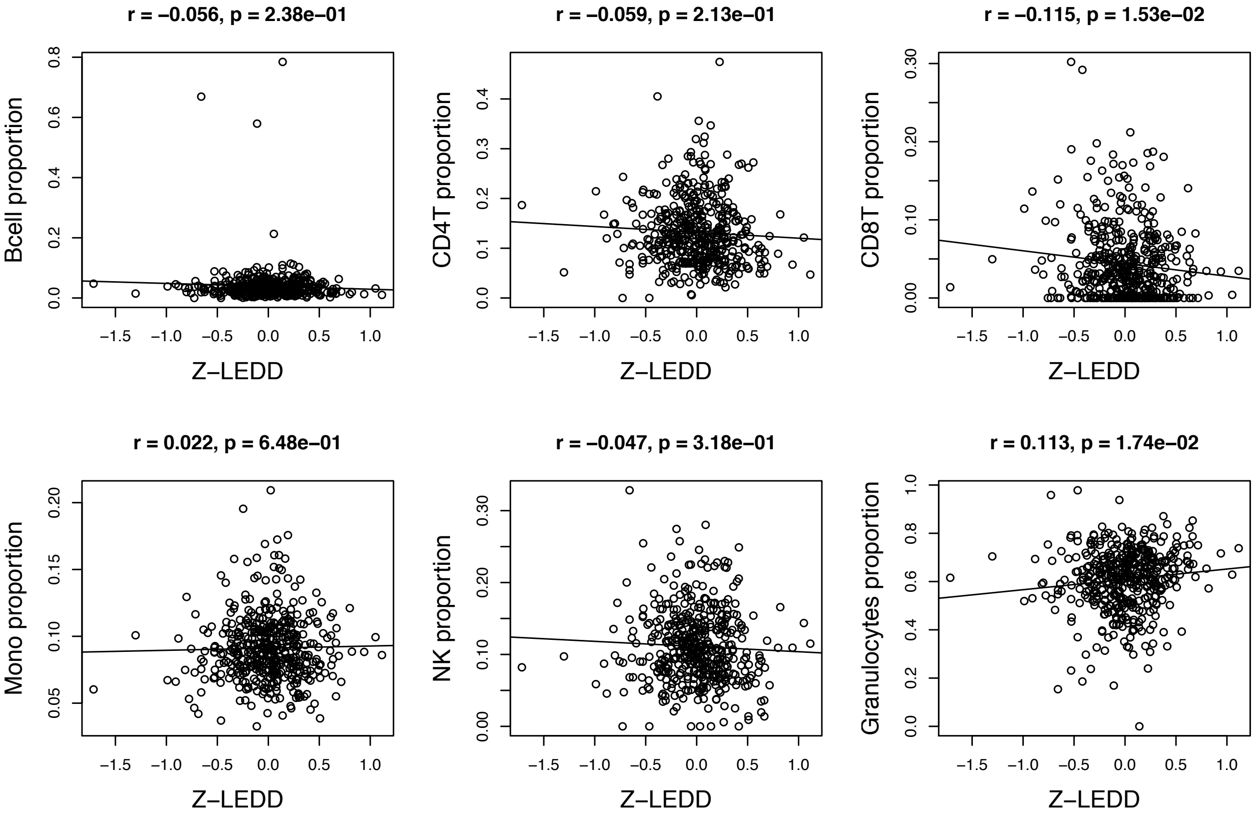

Supplementary Figure 2: Relationship (Pearson's correlations) between blood cell type proportions and Z-scaled LEDD after adjustment for disease duration in 445 PD cases from the SGPD dataset. Granulocytes are calculated as the sum of eosinophil and neutrophil proportions. NK, natural killer cells; Mono, monocytes.

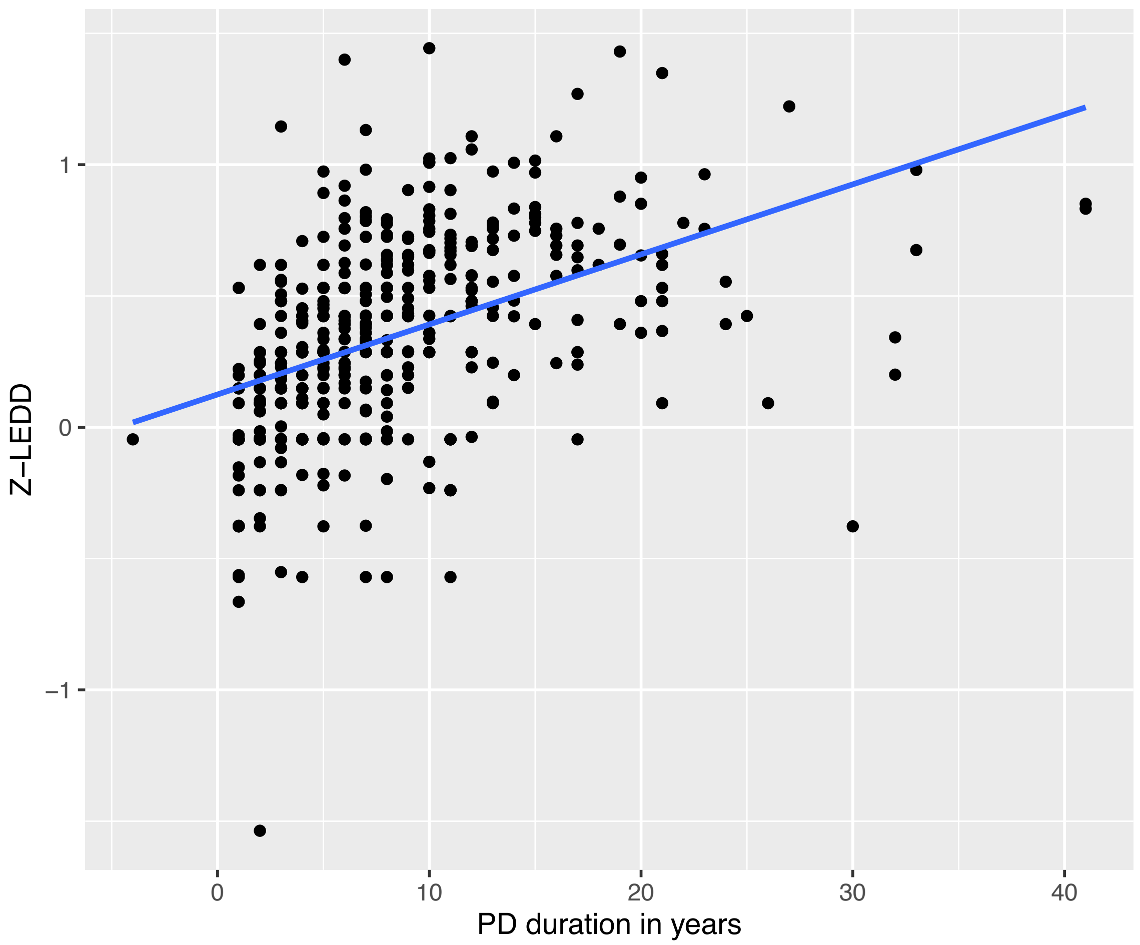

Supplementary Figure 3: Correlation between disease duration and Z-scaled LEDD in 445 PD cases from the SGPD dataset (Pearson's correlation = 0.44, p = 5.8×10^{-23}).

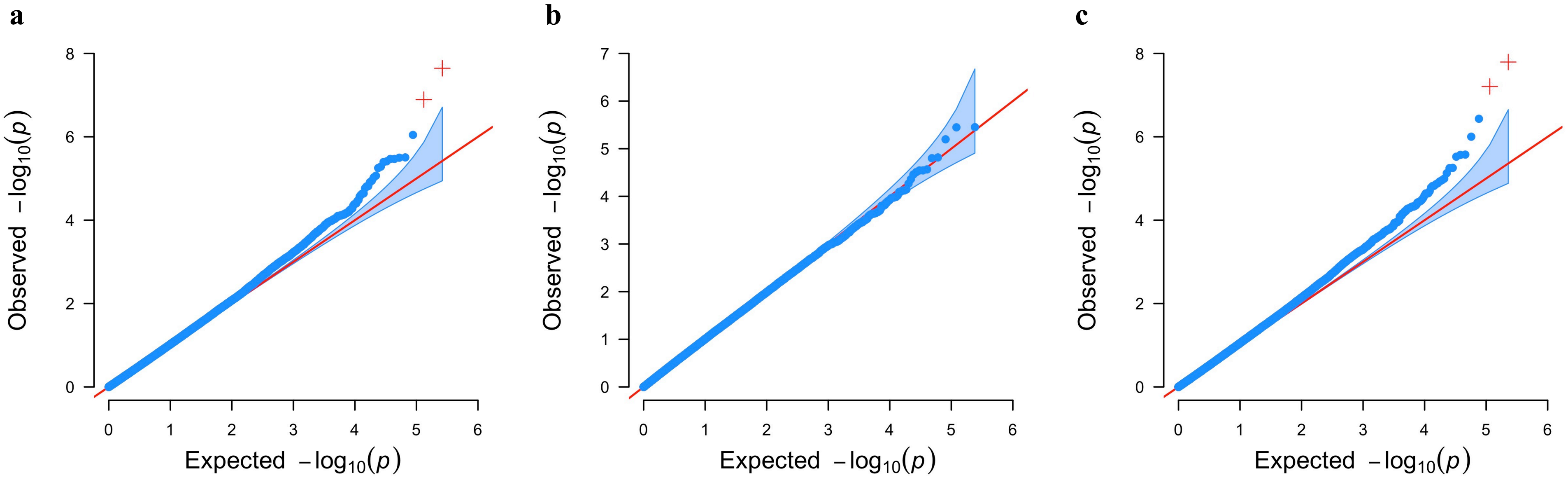

Supplementary Figure 4: Quantile-quantile (Q-Q) plots of the MOA MWAS for PD in the (a) SGPD cohort (N = 1,638 unrelated Europeans, N = 851 PD cases, N = 787 controls), (b) PEG cohort (N = 493 Europeans, N = 281 PD cases, N = 212 controls), and (c) meta-analysis of SGPD and PEG (N = 2,131 Europeans, N = 1,132 PD cases, N = 999 controls).

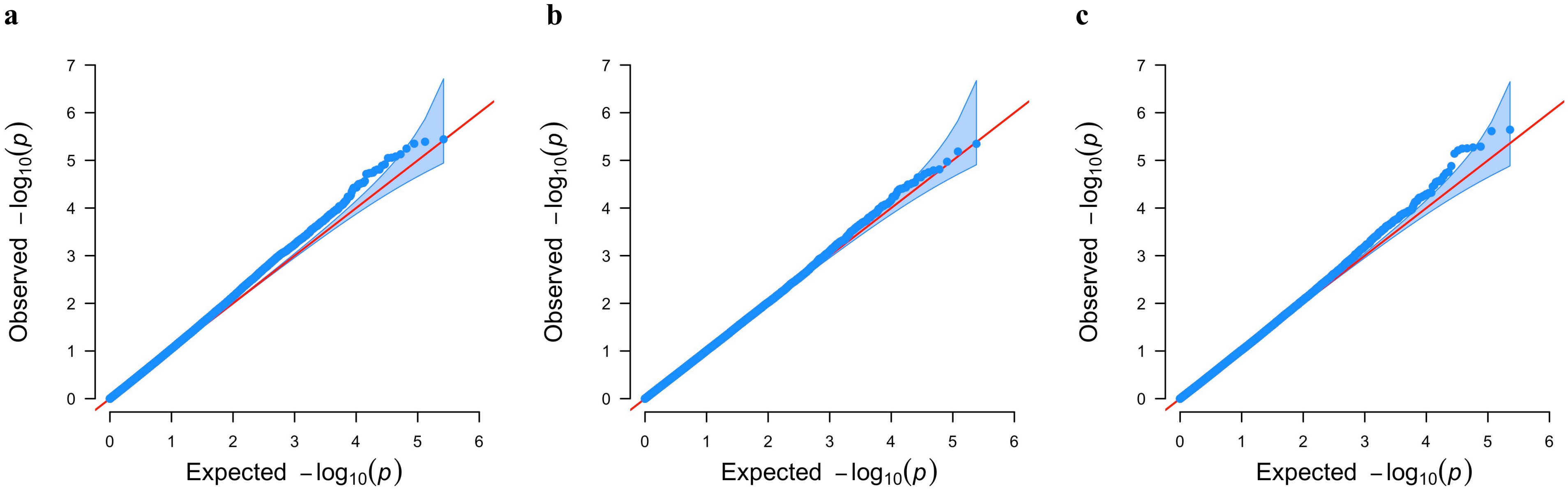

Supplementary Figure 5: Quantile-quantile (Q-Q) plots of the MOMENT MWAS for PD in the (a) SGPD cohort (N = 1,638 unrelated Europeans, N = 851 PD cases, N = 787 controls), (b) PEG cohort (N = 493 Europeans, N = 281 PD cases, N = 212 controls), and (c) meta-analysis of SGPD and PEG (N = 2,131 Europeans, N = 1,132 PD cases, N = 999 controls).

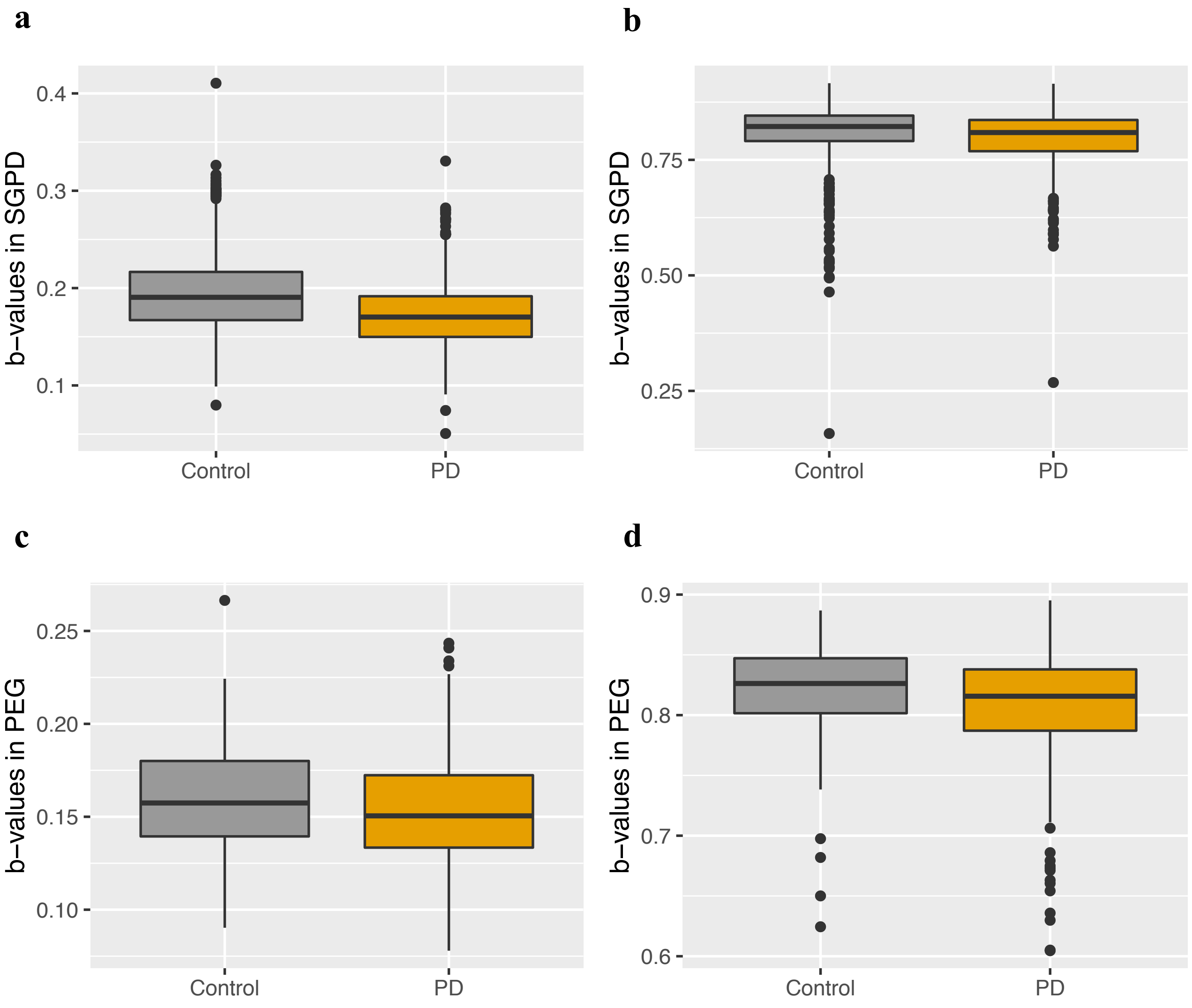

Supplementary Figure 6: Distribution of DNA methylation B values in PD cases and controls for epigenome-wide significant CpG probes identified in the MOA MWAS of SGPD. Panels show B values for (a) cg16001422 in SGPD, (b) cg26033520 in SGPD, (c) cg16001422 in PEG, (d) cg26033520 in PEG. Box plot center lines show the median, box limits denote upper and lower quartiles, whiskers represent 1.5x interquartile range and individual points show outliers.

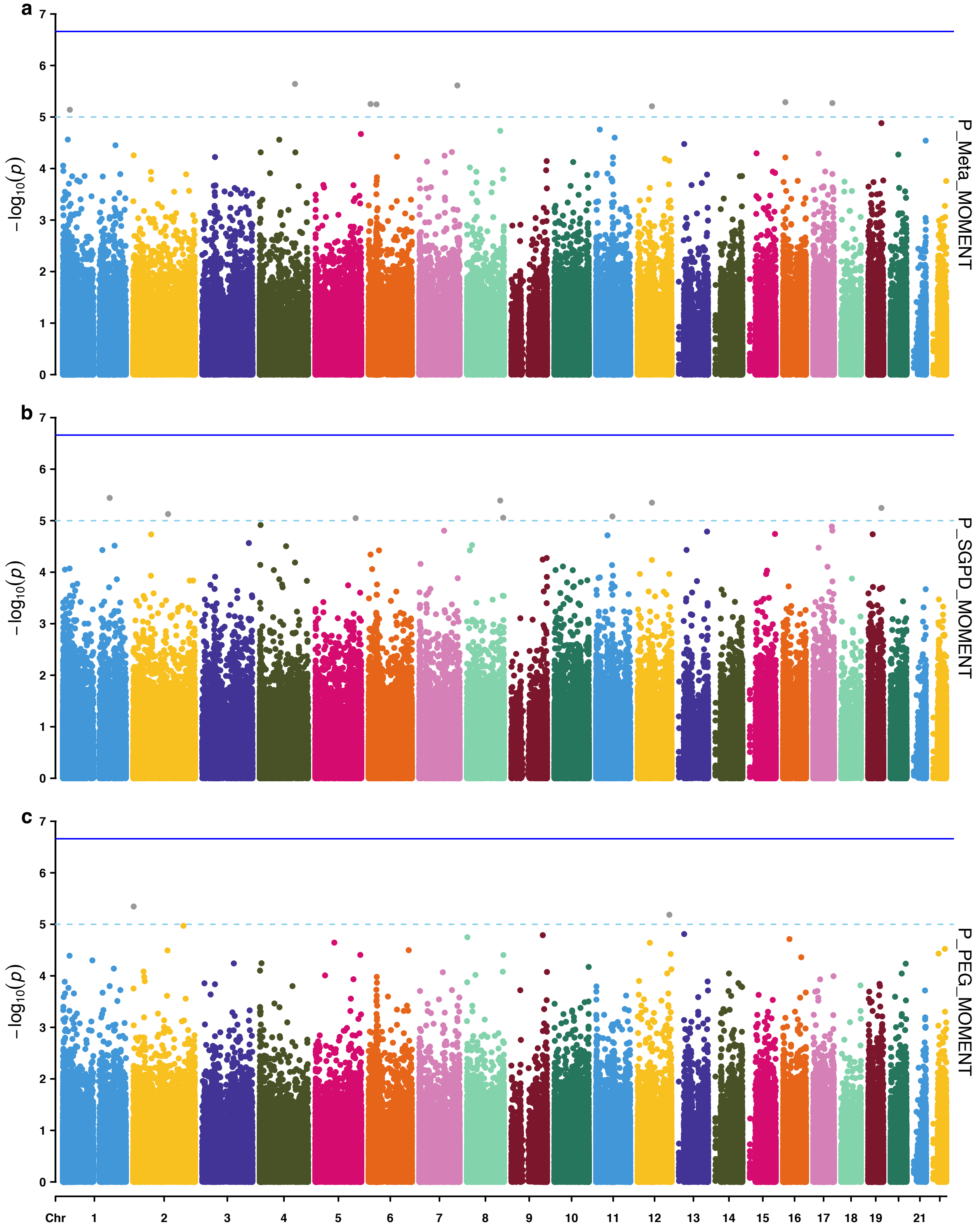

Supplementary Figure 7: Manhattan plots of the MOMENT MWAS of PD. (a) MOMENT MWAS meta-analysis of PD in the SGPD and PEG cohorts (N = 2,131 individuals); (b) MOMENT MWAS of PD in the SGPD dataset (N = 1,638 individuals); (c) MOMENT MWAS for PD in the PEG dataset (N = 493 individuals).

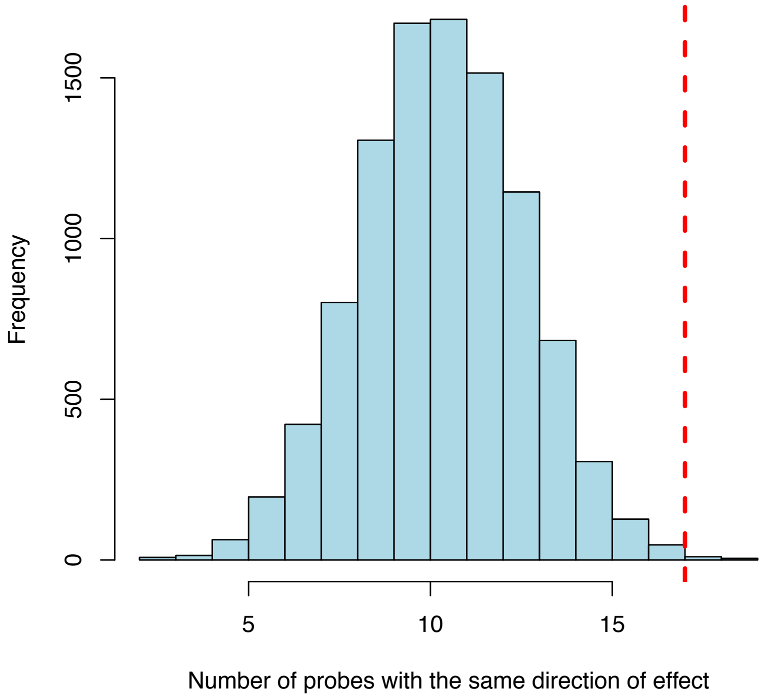

Supplementary Figure 8: Empirical distribution of the number of probes with the same direction of effect in the discovery (SGPD) and replication (PEG) cohorts based on 10,000 random samples of 21 probes. The red dashed line indicates our observation of 17 out of 21 approximately independent probes (R^2<0.1) with SGPD MOA $p < 1x10^{-04}$ with the same direction of effect in the PEG MOA MWAS.

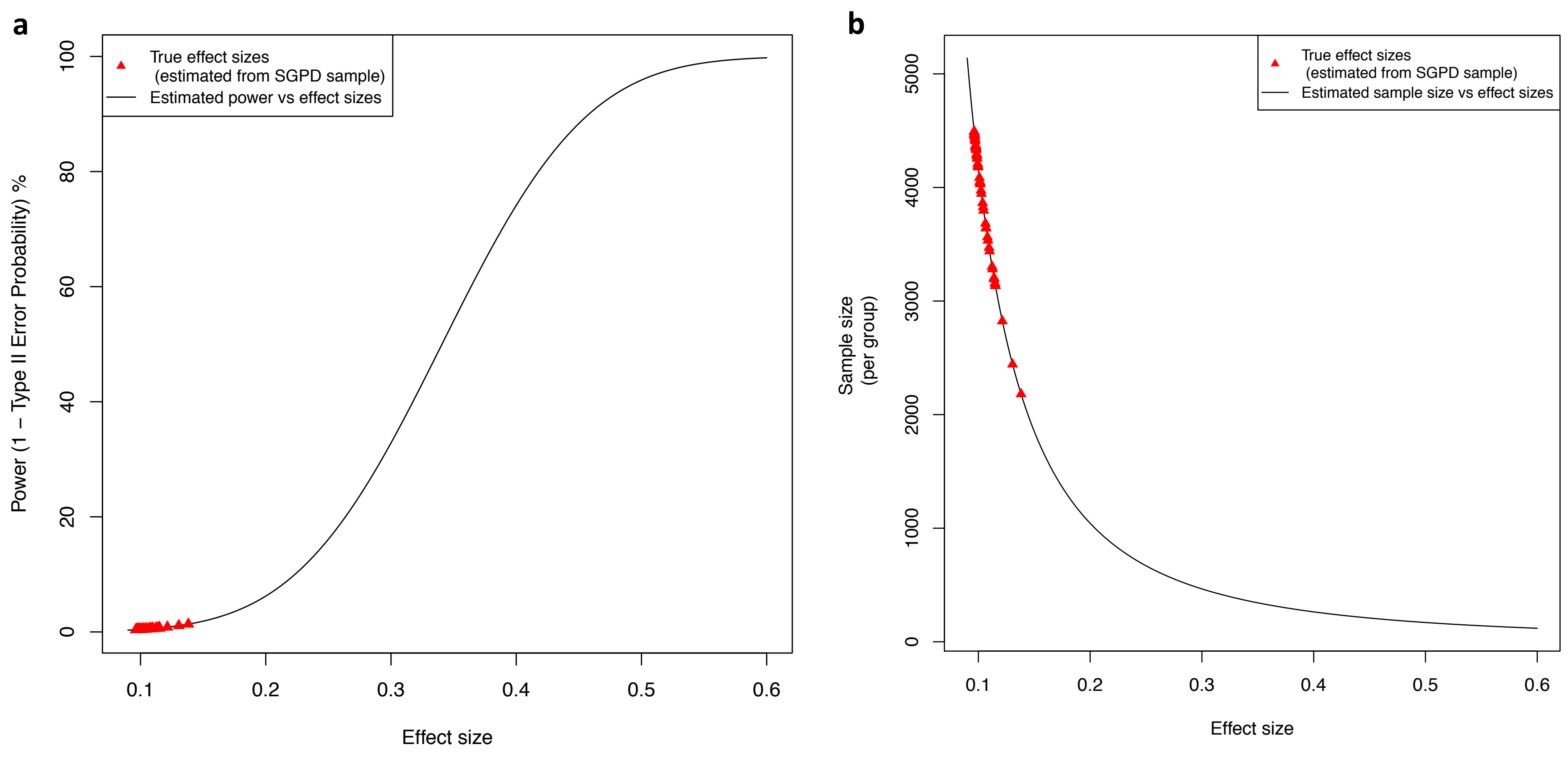

Supplementary Figure 9: Post-hoc statistical power calculations for replication of probes identified in SGPD at p < 1 x 10^{-04}. (a) Power to replicate CpG associations in the PEG cohort (281 PD cases, 212 controls) as a function of effect size, at a significance threshold of p = 9.1x10^{-04} (0.05/55). (b) Sample size (y-axis) necessary to identify a true association with the corresponding effect sizes (x-axis), based on pre-determined 80% power and replication significance threshold p-value = 9.1x10^{-04}. Red triangles represent the true (absolute) effect sizes of the 55 probes with SGPD p-value < 1x10^{-04} that are shared between the two datasets. Specifically, true effect sizes are calculated as absolute values of $b\ /(\sqrt{N_{eff}}\ \times\ se)$, where ***b*** is the estimated effect size of each probe on the phenotype, ***se*** is its standard error and N_{eff} = 4 * (N SGPD cases * N SGPD controls) / (N SGPD cases + N SGPD controls).

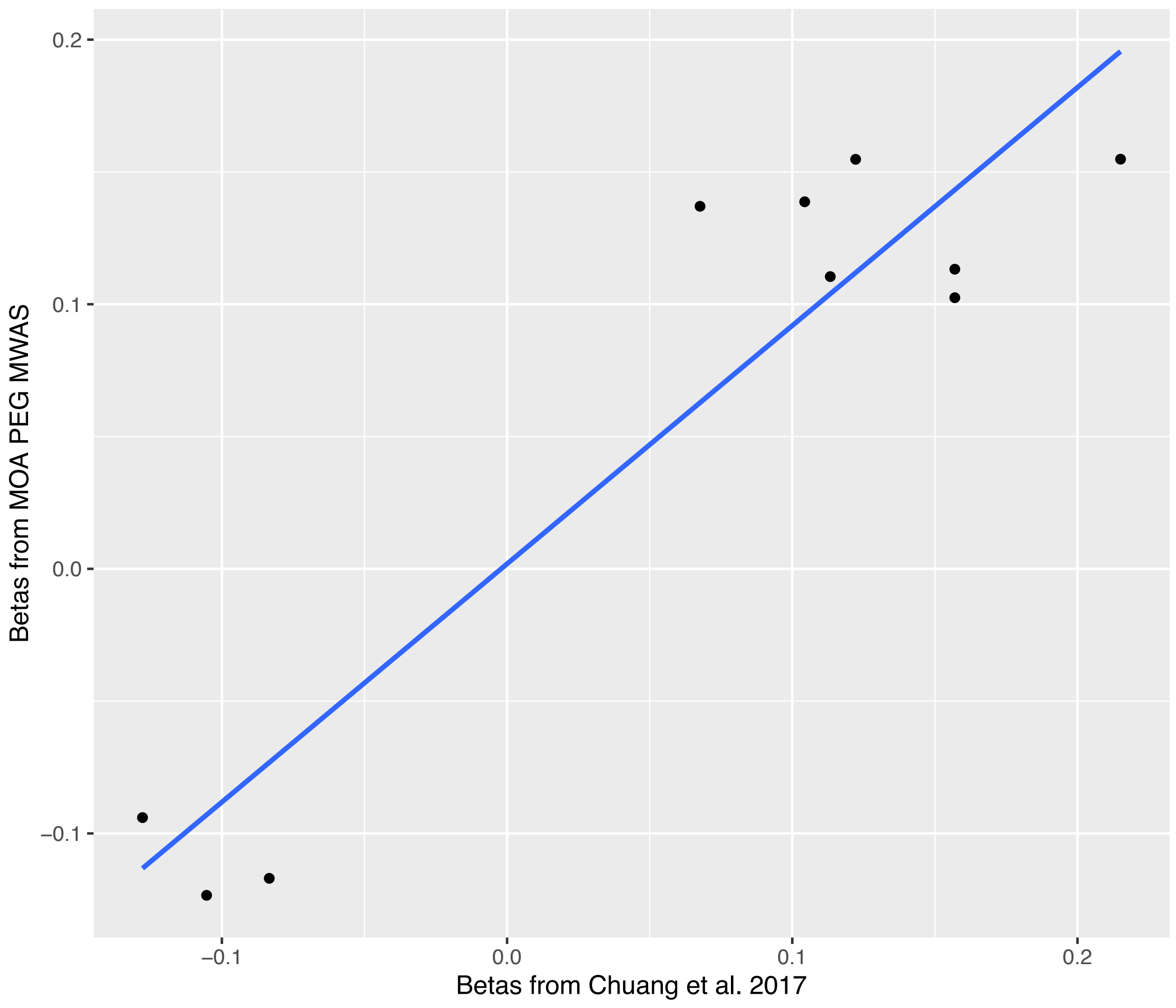

Supplementary Figure 10: Correlation of effect sizes between Chuang *et al.* and our MOA MWAS of PEG for the 10 (of 19) shared probes identified by Chuang *et al.* at $p < 5x10^{-06}$ in their analysis adjusted for CTPs (Pearson's correlation = 0.93, $p = 8.9x10^{-05}$). Of note, effect sizes from our MOA MWAS of PEG data were standardized using the following formula: $b_{std} = b\ /(\sqrt{N_{eff}} \times se)$, where ***b*** is the estimated effect size of each probe on the phenotype, ***se*** is its standard error and N_{eff} = 4 * (N PEG cases * N PEG controls) / (N PEG cases + N PEG controls).

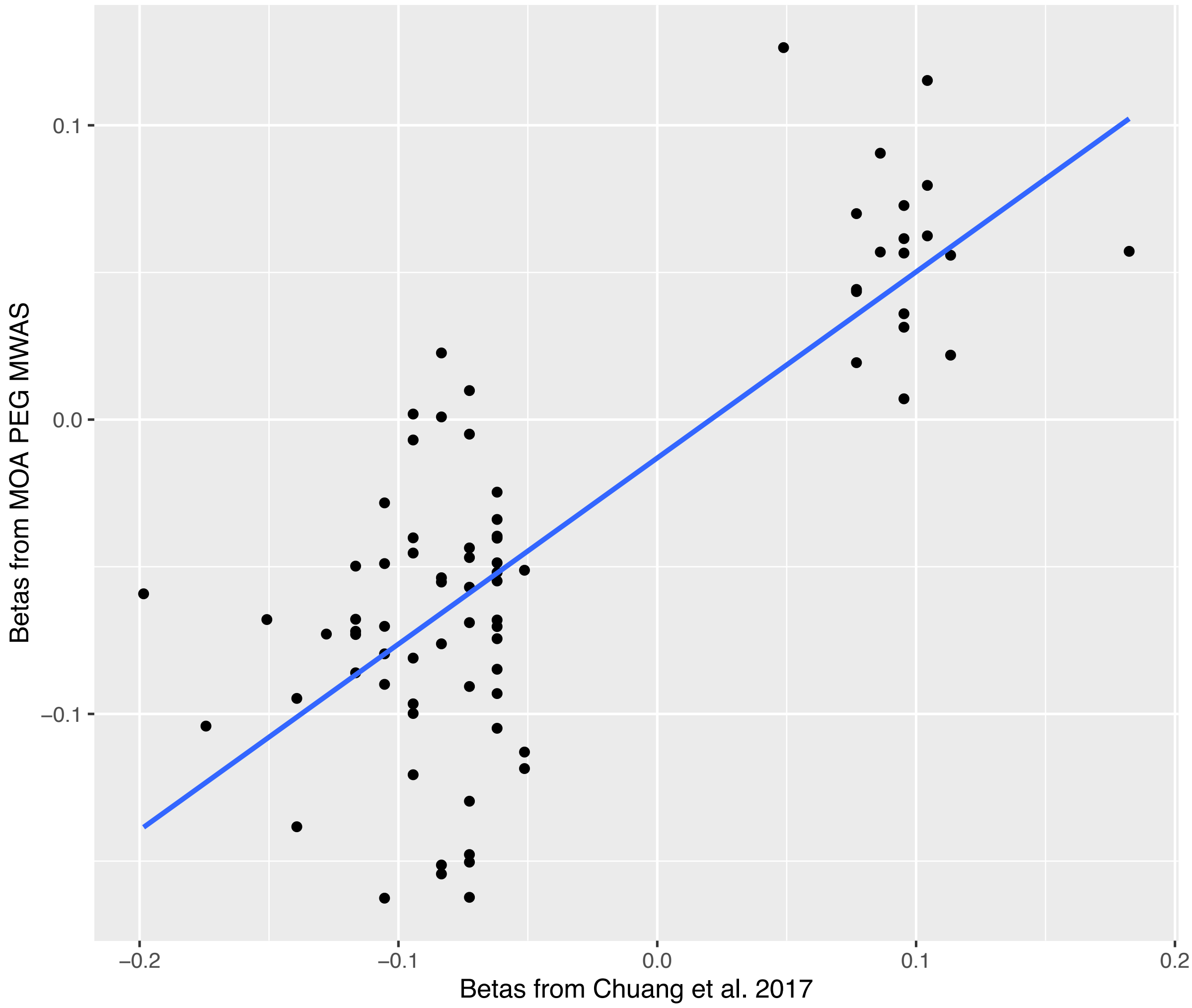

Supplementary Figure 11: Correlation of effect sizes between Chuang *et al.* and our MOA MWAS of PEG for the 78 (of 82) shared genome-wide significant probes identified by Chuang *et al.* in their analysis not adjusted for CTPs (Pearson's correlation = 0.77, $p < 2.2 \times 10^{-16}$). Of note, effect sizes from our MOA MWAS of PEG data were standardized using the following formula: $b_{std} = b\,/(\sqrt{N_{eff}} \times se)$, where ***b*** is the estimated effect size of each probe on the phenotype, ***se*** is its standard error and N_{eff} = 4 * (N PEG cases * N PEG controls) / (N PEG cases + N PEG controls).

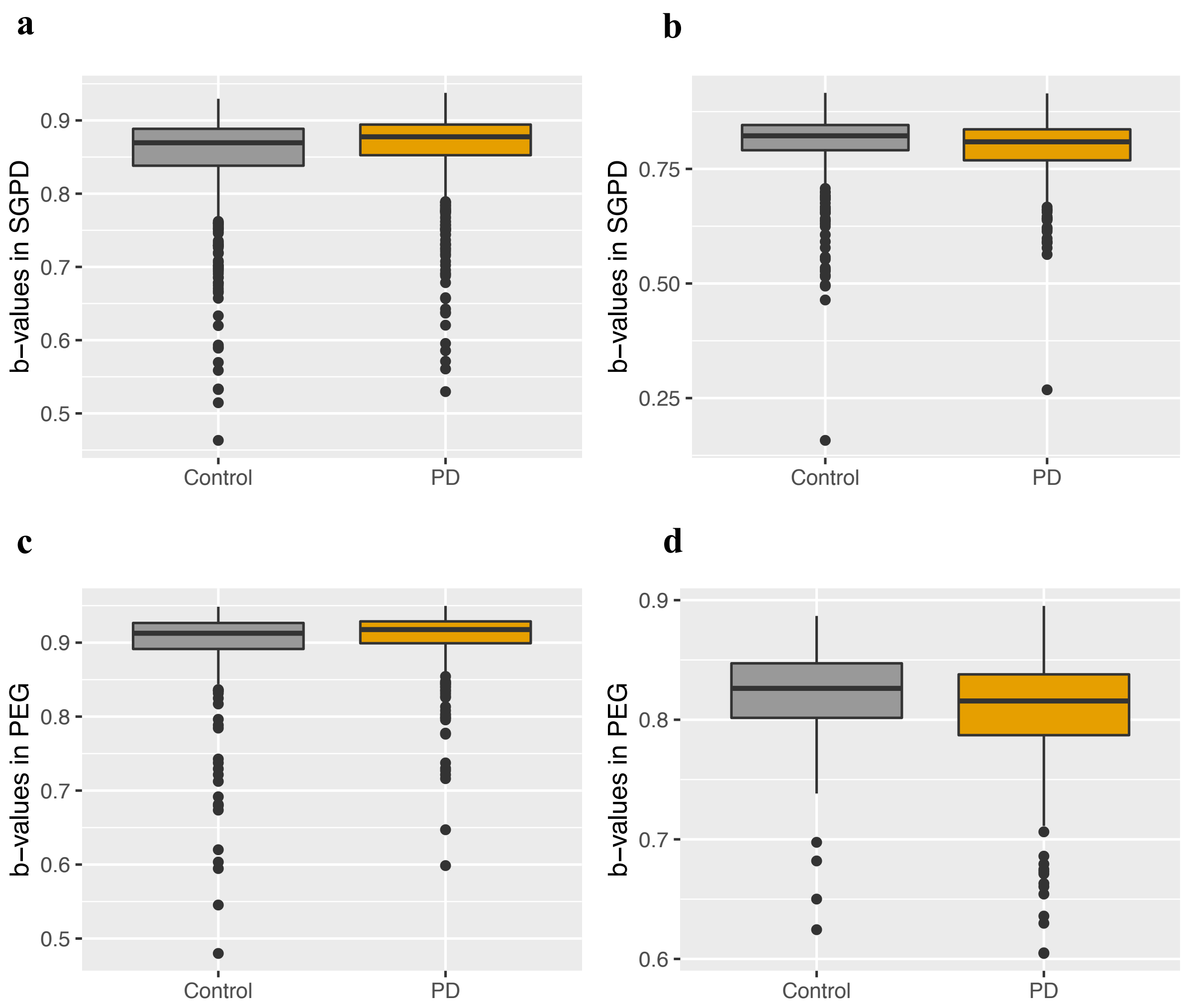

Supplementary Figure 12: Distribution of DNA methylation B values in PD cases and controls for epigenome-wide significant CpGs identified in the MOA MWAS meta-analysis of SGPD and PEG. Panels show B values for (a) cg06690548 in SGPD, (b) cg26033520 in SGPD, (c) cg06690548 in PEG, (d) cg26033520 in PEG. Box plot center lines show the median, box limits denote upper and lower quartiles, whiskers represent 1.5x interquartile range and individual points show outliers.

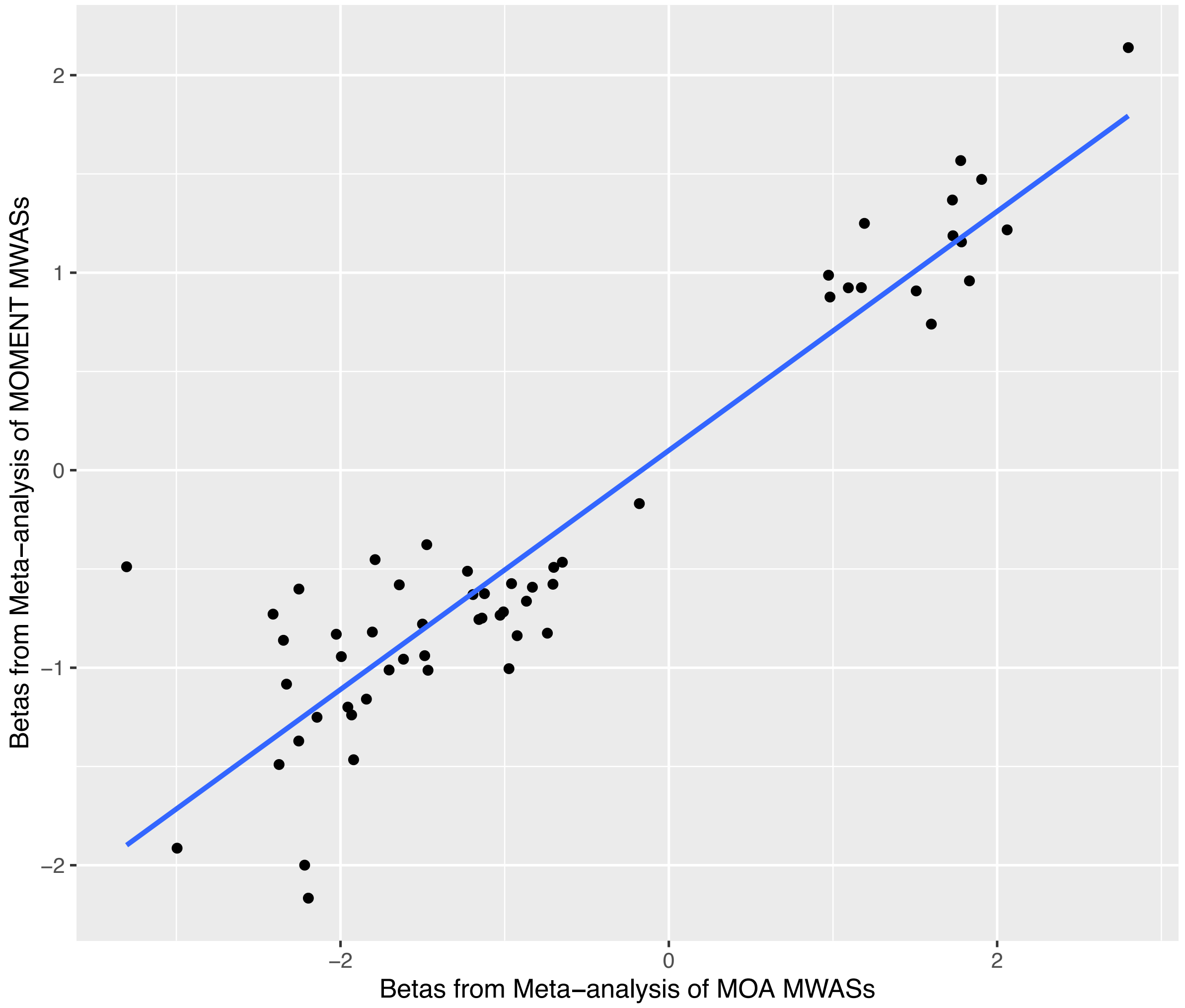

Supplementary Figure 13: Correlation of effect sizes of CpG's with meta-analysis $p_{MOA} < 1x10^{-04}$ (N=58) in the MOA and MOMENT meta-analysis of SGPD and PEG MWAS's (Pearson's correlation = 0.93, $p < 2.2x10^{-16}$).
