# Supplementary material for: Analysis of DNA methylation associates the cystine–glutamate antiporter SLC7A11 with risk of Parkinson’s disease
Source: Nat Commun. 2020 Mar 6;11:1238. doi: 10.1038/s41467-020-15065-7 (PMC7060318; doi:10.1038/s41467-020-15065-7)
Supplement: Supplementary file 7 — Reporting Summary [file 41467_2020_15065_MOESM7_ESM.pdf]

nature research

Corresponding author(s): Jacob Gratten, Peter Visscher

Last updated by author(s): 2019/12/19

Reporting Summary

Nature Research wishes to improve the reproducibility of the work that we publish. This form provides structure for consistency and transparency in reporting. For further information on Nature Research policies, see Authors & Referees and the Editorial Policy Checklist .

Statistics

For all statistical analyses, confirm that the following items are present in the figure legend, table legend, main text, or Methods section.

n/a	Confirmed	
☐	☒	The exact sample size (*n*) for each experimental group/condition, given as a discrete number and unit of measurement
☐	☒	A statement on whether measurements were taken from distinct samples or whether the same sample was measured repeatedly
☐	☒	The statistical test(s) used AND whether they are one- or two-sided<br>*Only common tests should be described solely by name; describe more complex techniques in the Methods section.*
☐	☒	A description of all covariates tested
☐	☒	A description of any assumptions or corrections, such as tests of normality and adjustment for multiple comparisons
☐	☒	A full description of the statistical parameters including central tendency (e.g. means) or other basic estimates (e.g. regression coefficient) AND variation (e.g. standard deviation) or associated estimates of uncertainty (e.g. confidence intervals)
☐	☒	For null hypothesis testing, the test statistic (e.g. *F*, *t*, *r*) with confidence intervals, effect sizes, degrees of freedom and *P* value noted<br>*Give P values as exact values whenever suitable.*
☒	☐	For Bayesian analysis, information on the choice of priors and Markov chain Monte Carlo settings
☒	☐	For hierarchical and complex designs, identification of the appropriate level for tests and full reporting of outcomes
☐	☒	Estimates of effect sizes (e.g. Cohen's *d*, Pearson's *r*), indicating how they were calculated

Our web collection on statistics for biologists contains articles on many of the points above.

Software and code

Policy information about availability of computer code

Data collection	Raw Illumina Human Methylation 450K BeadChip data was processed using recommended protocols in the Genome Studio software.
Data analysis	The meffil package in the R statistical computing software was used for data quality control (QC) and to impute blood cell type proportions from the QC'ed methylation data. The Omics-data-based Complex trait Analysis (OSCA) software was used to perform additional QC and for genome-wide mixed model-based association testing.

For manuscripts utilizing custom algorithms or software that are central to the research but not yet described in published literature, software must be made available to editors/reviewers. We strongly encourage code deposition in a community repository (e.g. GitHub). See the Nature Research guidelines for submitting code & software for further information.

Data

Policy information about availability of data

All manuscripts must include a data availability statement. This statement should provide the following information, where applicable:

- Accession codes, unique identifiers, or web links for publicly available datasets
- A list of figures that have associated raw data
- A description of any restrictions on data availability

The SGPD methylation data is available from the Gene Expression Omnibus (GEO) [https://www.ncbi.nlm.nih.gov/geo/query/acc.cgi?acc=XXXXXXXXX]. The SGPD genetic relationship matrix (GRM) and top two genetic principal components, used to identify unrelated European individuals for inclusion in DNA methylation analyses, are available from cnsgenomics.com/data/vallerga_et_al_2020_nc. The SGPD MWAS summary statistics are also available from cnsgenomics.com/data/vallerga_et_al_2020_nc. The PEG methylation data is available from GEO [https://www.ncbi.nlm.nih.gov/geo/query/acc.cgi?acc=GSE111629].

Field-specific reporting

Please select the one below that is the best fit for your research. If you are not sure, read the appropriate sections before making your selection.

☒ Life sciences ☐ Behavioural & social sciences ☐ Ecological, evolutionary & environmental sciences

For a reference copy of the document with all sections, see nature.com/documents/nr-reporting-summary-flat.pdf

Life sciences study design

All studies must disclose on these points even when the disclosure is negative.

Sample size	Sample size in the SGPD cohort was determined by the number of samples available for DNA methylation profiling, rather than a power analysis assuming a specific effect size for disease-associated probes. However, the SGPD sample is larger than any previously published study of DNA methylation in Parkinson's disease (PD), some of which have reported PD-associated methylation probes.
Data exclusions	We performed stringent quality control on both the SGPD and PEG datasets to exclude poor quality probes and samples, as described in the Methods. Additionally, analyses were restricted to the sub-set of unrelated European individuals in each cohort.
Replication	The PEG cohort was used as an independent replication sample for epigenome-wide significant associations identified in the SGPD discovery sample.
Randomization	Randomization of individuals was not performed. Individuals were assigned to groups according to whether they had received a diagnosis of PD. A high proportion of controls (>50%) in the SGPD cohort were randomly selected community-based volunteers matched to patients on the basis of age, post code (i.e. geographic location) and ancestry.
Blinding	We did not use blinding because this wasn't appropriate for the analyses performed in the study.

Reporting for specific materials, systems and methods

We require information from authors about some types of materials, experimental systems and methods used in many studies. Here, indicate whether each material, system or method listed is relevant to your study. If you are not sure if a list item applies to your research, read the appropriate section before selecting a response.

Materials & experimental systems

n/a	Involved in the study
☒	☐ Antibodies
☒	☐ Eukaryotic cell lines
☒	☐ Palaeontology
☒	☐ Animals and other organisms
☐	☒ Human research participants
☒	☐ Clinical data

Methods

n/a	Involved in the study
☒	☐ ChIP-seq
☒	☐ Flow cytometry
☒	☐ MRI-based neuroimaging

Human research participants

Policy information about studies involving human research participants

Population characteristics	The SGPD cohort includes PD cases and age-matched controls from Australia and New Zealand. DNA samples were bisulphite converted and processed on Illumina Human Methylation 450K arrays in two batches.
Recruitment	The SGPD cohort comprises PD cases and controls recruited by the Queensland Parkinson's Project (QPP), the New Zealand Brain Research Institute (NZBRI) and Parkinson's Disease Research Clinic at the Brain and Mind Research Institute, University of Sydney.
Ethics oversight	Ethical approval for the study was obtained from the University of Queensland Human Research Ethics Committee (2011001173), the Griffith University Human Research Ethics Committee (ESK0411HREC), the Southern Health and Disability Ethics committee (New Zealand: URA/11/08/042; URB/09/08/037) and the University of Sydney Human Research Ethics Committee (10963; 2013/945).

Note that full information on the approval of the study protocol must also be provided in the manuscript.
