# Supplementary material for: Analysis of DNA methylation associates the cystine–glutamate antiporter SLC7A11 with risk of Parkinson’s disease
Source: Nat Commun. 2020 Mar 6;11:1238. doi: 10.1038/s41467-020-15065-7 (PMC7060318; doi:10.1038/s41467-020-15065-7)
Supplement: Supplementary file 8 — Description of Additional Supplementary Files [file 41467_2020_15065_MOESM8_ESM.pdf]

Title: Supplementary Data 1

Description: Summary results for CpG probes with $p \leq 1 \times 10^{-3}$ from the MOA and MOMENT SGPD MWAS analyses. The masking columns “MASK.snp5.GMAF1p” and “MASK.snp5.common” denote whether the CpG probe 5bp 3'-subsequence (including extension for type II) overlaps with any SNP with global MAF >1%, or with any common SNP in dbSNP, including those with global MAF <1%, respectively. “Probe_Start” and “Probe_End” denote the mapped start and end position of the probe (50bp long), based on the hg19 Human Methylation 450K annotation file.

Title: Supplementary Data 2

Description: Summary results for CpG probes with $p \leq 1 \times 10^{-3}$ from the MOA and MOMENT PEG MWAS analyses. The masking columns “MASK.snp5.GMAF1p” and “MASK.snp5.common” denote whether the CpG probe 5bp 3'-subsequence (including extension for type II) overlaps with any SNP with global MAF >1%, or with any common SNP in dbSNP, including those with global MAF <1%, respectively. “Probe_Start” and “Probe_End” denote the mapped start and end position of the probe (50bp long) based on the hg19 Human Methylation 450K annotation file.

Title: Supplementary Data 3

Description: Summary results for CpG probes with $p \leq 1 \times 10^{-3}$ from the MOA and MOMENT MWAS meta-analyses of SGPD and PEG. The masking columns “MASK.snp5.GMAF1p” and “MASK.snp5.common” denote whether the CpG probe 5bp 3'-subsequence (including extension for type II) overlaps with any SNP with global MAF >1%, or with any common SNP in dbSNP, including those with global MAF <1%, respectively. “Probe_Start” and “Probe_End” denote the mapped start and end position of the probe (50bp long) based on the hg19 Human Methylation 450K annotation file.

Title: Supplementary Data 4

Description: MOA and MOMENT MWAS meta-analyses summary results for CpG probes in known PD genes.
